# LncRNA CERS6-AS1 promotes proliferation and metastasis through the upregulation of YWHAG and activation of ERK signaling in pancreatic cancer

**DOI:** 10.1038/s41419-021-03921-3

**Published:** 2021-06-24

**Authors:** Jian Xu, Jie Wang, Zhiwei He, Peng Chen, Xueyi Jiang, Yankun Chen, Xinyuan Liu, Jianxin Jiang

**Affiliations:** 1 Department of Hepatobiliary Surgery, Renmin Hospital of Wuhan University, Wuhan, China; 2 Department of Hepatic-Biliary-Pancreatic Surgery, The Affiliated Hospital of Guizhou Medical University, Guiyang, China

**Keywords:** Oncogenes, Tumour biomarkers

## Abstract

LncRNAs play essential regulatory roles in pancreatic cancer (PC) tumorigenesis and progression. We aimed to investigate the role of lncRNA CERS6-AS1 in PC. CERS6-AS1 expression was determined in PC tissues and cell lines by PCR analysis. The roles of CERS6-AS1 on proliferation, migration, invasion, and epithelial to mesenchymal transition (EMT) were confirmed via CCK-8 assay, EDU assay, transwell assay, wound healing assay, and western blot assay. Besides, the interaction between CERS6-AS1 and their target genes was verified by luciferase report assays and RIP assays. Animal assays and clinical data analysis were performed to validate the functions in vivo. We found that lncRNA CERS6-AS1 was highly expressed in PC tissues and cells. Additionally, high expression of CERS6-AS1 was obviously associated with poor prognosis. Functional assays demonstrated that CERS6-AS1 downregulation significantly inhibited PC cell growth and migration. Moreover, CERS6-AS1 exerted as a molecular sponge for miR-217-5p (miR-217), and miR-217 was confirmed as a potential target of CERS6-AS1. Subsequently, miR-217 suppressed PC cell proliferation and metastasis by directly targeting YWHAG, which interacted with RAF1 and promoted its phosphorylation, leading to RAF1-mediated ERK signaling activation and translocation of phosphorylated ERK from the cytoplasm to the nucleus. Mechanically, CERS6-AS1 silencing significantly inhibited PC cell proliferation and metastasis via a miR-217/YWHAG/RAF1 signaling axis. CERS6-AS1 exerts as a carcinogen in PC to promote malignant features and behaves as a competitive endogenous RNA for miR-217. We identified CERS6-AS1 as a potential biomarker or therapeutic target to improve PC diagnosis and treatment outcomes.

## Introduction

Pancreatic cancer (PC) is a highly malignant digestive cancer with a high mortality rate and a 5-year survival rate of less than 10% [1]. Although significant improvements have been achieved in surgery and radiochemotherapy, the recurrence and mortality rates are still high [2]. Currently, radical resection remains the most effective treatment for PC. However, patients who suffer from PC often have less obvious symptoms in the early stages, leading to lost opportunities for surgical treatment due to arteriovenous invasion or distant metastasis [3]. Therefore, there is an urgent need to elucidate the underlying molecular mechanism of PC and explore effective biomarkers and novel therapeutic targets to achieve better-individualized treatment and improve the prognosis of PC patients.

Long non-coding RNAs (lncRNAs) are defined as a class of non-protein-coding transcripts that are more than 200 nucleotides in length. They are involved in multiple biological processes, such as cell differentiation, proliferation, apoptosis, angiogenesis, and others [4]. Accumulated evidence has demonstrated that the aberrant expression of lncRNAs is significantly associated with cancer progression. For instance, the downregulated expression of lncRNA H19 promoted erlotinib resistance via the upregulation of PKM2 and activation of AKT signaling in EGFR-mutant lung cancer [4]. In addition, LINC01232 overexpression in PC tissues promoted PC metastasis by inhibiting the ubiquitin-mediated degradation of HNRNPA2B1, which was reported to be involved in the alternative splicing of A-Raf [5]. Moreover, lncRNA FLVCR1-AS1 functioned as an oncogene to promote ovarian cancer cell progression, metastasis, and epithelial to mesenchymal transition (EMT) by competitively binding to miR-513 to activate YAP1 signaling [6]. Conversely, Qin et al suggested that lncRNA LINC00657 inhibited cervical cancer progression by functioning as a competitive endogenous RNA (ceRNA) and sponging miR-20a-5p [7]. This prevented miR-20a-5p from binding and suppressing RUNX3 mRNA, thereby enhancing RUNX3/DR5-mediated NK cell susceptibility [7]. LncRNA CERS6-AS1 was reported to exert oncogenic functions in breast cancer [8] and hepatocellular carcinoma [9]{#1}. However, the function and underlying molecular mechanism of CERS6-AS1 in PC remain poorly understood.

MicroRNAs (miRNAs), a well-known group of small non-coding RNAs, exert their regulatory roles in mRNA degradation or post-transcriptional gene silencing through directly binding the 3′-untranslated region (UTR) of target mRNAs [10]. In-depth studies have demonstrated that lncRNAs play essential roles in malignant tumors by acting as ceRNAs to sponge miRNAs. The lncRNAs with this function can adsorb miRNAs and indirectly release the inhibitory effects of miRNAs on their target genes [11].

In this study, we aimed to elucidate the function and underlying molecular mechanism of CERS6-AS1 in PC progression. Interestingly, we found that CERS6-AS1 was significantly overexpressed in PC tissues/cells and positively correlated with malignant clinicopathologic features and a poor prognosis. CERS6-AS1 knockdown inhibited cell proliferation and metastasis in vitro and in vivo. Further investigation revealed that CERS6-AS1 increased the expression of YWHAG by sponging miR-217-5p (miR-217), resulting in the phosphorylation of RAF1 and activation of ERK signaling. Therefore, we identified a novel potential diagnostic and therapeutic target.

## Materials and methods

### Tissue specimens

Twenty-nine pairs of PC and adjacent non-tumor samples were collected from Renmin Hospital of Wuhan University from 2016 to 2020. Fresh samples were preserved in liquid nitrogen. All samples were collected with patient consent and signed informed consent. This study was approved by the Ethics Committee of Renmin Hospital of Wuhan University.

### Cell culture

Two PC cell lines (AsPC-1 and BxPC-3) and the normal human pancreatic ductal epithelial cell line (HPDE) were cultured in an incubator (37 °C, 5%CO_2_) in RPMI 1640 medium (Hyclone, USA) supplemented with 10% fetal bovine serum (FBS) (Gibco, USA). Another four PC cell lines (Capan-2, SW1990, PANC-1, and MIA PaCa-2) were cultured in DMEM (Hyclone, USA) supplemented with 10% FBS (Gibco, USA). All cell lines were purchased from the American Type Culture Collection.

### Cell transfection and transduction

Vectors encoding YWHAG (YWHAG), YWHAG silencing RNA (si-YWHAG), and their paired control was purchased from Ribobio (Guangzhou, China). Besides, miR-217 overexpression (miR-217 mimic), overexpressed control (miR-217 NC), miR-217 knockdown (miR-217 inhibitor), and knockdown control (miR-217 inhibitor NC) were also purchased from Ribobio. CERS6-AS1 silencing sequences (shCERS6-AS1-1 and shCERS6-AS1-2) and the control sequence (shCtrl) were respectively cloned and stably expressed in lentiviruses which were purchased from Genechem (Shanghai, China). Cell transfections and transduction were carried out in accordance with the manufacturer’s protocol.

### qRT-PCR analysis

Total RNA from PC tissues or cells was extracted using TRIzol (Invitrogen, USA) in accordance with the manufacturer’s protocol. The HiScript® III 1st Strand cDNA Synthesis Kit (+gDNA wiper) (Vazyme, China) was performed to reverse transcription of the common gene, and the miRNA First Strand cDNA Synthesis Kit (by stem-loop) (Vazyme, China) was performed to reverse transcription of miRNA. The target genes were quantified by qPCR amplification using the ChamQ Universal SYBR qPCR Master Mix (Vazyme). GAPDH or U6 was used as the internal controls. The 2^−ΔΔCt^ method was applied for evaluating the levels of target gene amplification. Supplementary Table 1 showed all details of PCR primer sequences in this study.

### Cell proliferation assays

For CCK-8 assays, each of 96-well plates cultured PC cells transfected with silencing or control sequence of CERS6-AS1 (*N* = 1 × 10^4^). To evaluate cell viability, a 10% CCK8 working solution (Dojindo, Japan) was prepared and 100 µl was added into each well with incubating at 37 °C for 2 h. The 450 nm absorbance was used to measure the relative cell viability.

For colony formation assays, each of 6-well plates cultured PC cells transfected with silencing or control sequence of CERS6-AS1 (*N* = 2 × 10^3^) at 37 °C for 2 weeks, and the medium was changed regularly. Subsequently, the number of colony formation was evaluated after a series of steps, including washing with PBS, fixing with 4% paraformaldehyde, staining with crystal violet, and photographing under a microscope.

For EDU assays, transfected cells were cultured in the confocal dish (*N* = 5 10^5^). After washing and fixing, Triton (1%) was added to perforate cell membranes for 5 min. Cells were then incubated with the EDU dyeing agent for 30 min and stained with Hochest33258 for 5 min. Finally, a fluorescence microscope was used to capture the images. The EDU positive rate was evaluated by the ratio between the number of EDU positive cells(Cy3-probe) and the total number of cells(DAPI). The EDU reagent was purchased from Ribobio (Guangzhou, China).

### Cell migration and invasion assays

For wound healing assays, transfected cells were cultured in 6-well plates until confluence. The cell layer was then scratched with a sterile plastic tool. Cells were washed with PBS and imaged under a microscope every 24 h.

For transwell assays, the transwell chambers with a 6.5-mm, 8.0-μm-pore polycarbonate membrane were seeded with approximately 200 μl of serum-free medium containing 2 × 10^4^ cells. The 10% Matrigel (BD Biosciences, USA) was added into the upper chamber before the invasion assay. However, the 10% Matrigel (BD Biosciences, USA) was not necessary for migration assay. Approximately 800 μl DMEM supplemented with 20% FBS was filled with the lower chambers. Then, the transwell plates were incubated at 37 °C for 24 h. Subsequently, the number of cells on upper chambers was evaluated through a microscope after washing with PBS, fixing with 4% paraformaldehyde, and staining with 1% crystal violet.

### Tumor xenografts in nude mice

To evaluate tumorigenicity in vivo, we constructed a subcutaneous implantation model with stably expressed CERS6-AS1 silencing or control PC cells. Approximately 100 μl suspension containing 1 × 10^6^ cells was injected subcutaneously into the right armpit of mice under pentobarbital sodium anesthesia. The weights of nude mice and subcutaneous tumor volumes were recorded weekly. Subsequently, mice were executed without pain, and tumor tissues were harvested quickly and processed for further analysis, including PCR and immunohistochemical (IHC) analysis.

Then, we used transfected PC cells to establish a metastasis model to evaluate the metastatic ability in vivo. A median abdominal incision was made after pentobarbital sodium anesthesia. Cell suspensions (1 × 10^6^ cells in 100 μl PBS) were labeled with Luciferase and injected into the spleen of mice. The incision was sutured carefully to ensure that the mice woke up safely. In vitro imaging was performed 6 weeks later using an Imaging camera obscura platform. The mice were executed without pain after 2 months of feeding, and the livers were harvested by careful dissection. Subsequently, the number of metastases in livers was counted under a microscope.

### Western blot assays

The total protein in cells and tissues was extracted using RIPA buffer (Boster Biological Technology, China). Then the cell lysates were used for SDS-PAGE Electrophoresis System. And the proteins were transferred from polyacrylamide gel to the polyvinylidene fluoride (PVDF) membranes. The membranes were blocked by 5% bovine serum albumin (BSA) for 2 h. Subsequently, the membranes were added with primary antibodies, including the Phospho-Erk1/2 Pathway Sampler Kit (Item No:9911, containing p-ERK, p-MEK, p-RAF1, 1:500, Cell Signaling Technology, USA), YWHAG (1:500, Abcam, USA) and ERK, MEK, RAF1, GAPDH (1:1000, Abclonal, China), incubated at 4 °C for 10 h. Then, the membranes were washed with Tris Buffered Saline Tween (TBST) and incubated for 2 h at indoor temperature with a specific secondary antibody conjugated to horseradish peroxidase. Finally, the enhanced chemiluminescent Kit (Sangon, China) was used to expose the protein bands, and the gray values were analyzed by software.

### Luciferase reporter assays

The 3′-UTR sequence of miR-217 binding sites within the predicted target sites was amplified and cloned into the pmirGLO luciferase vector. PANC-1 or MIA PaCa-2 cells were cultured in 6-well plates until 50–70% confluence and then co-transfected with WT-pmirGLO (or MUT-pmirGLO) and mimic/inhibitor-miR-217 using Lipofectamine 3000. After 48 h, the luciferase activity was analyzed with the Dual-luciferase reporter assay system.

### RIP assay

The Magna RIP Assay Kit (Millipore, USA) was used for the RIP assay to evaluate the interaction of CERS6-AS1 with Ago2. The cells from different groups were lysed using complete RIP lysis buffer, and Magnetic beads conjugated with anti-Argonaute 2 (Ago2) or control anti-immunoglobulin G (IgG) antibodies were applied to incubate the cell extract. Then the cell extract was incubated at 4 °C for 8 h. After removing the protein beads, reverse transcription and qRT-PCR were performed.

### Immunofluorescence (IF) assays

First, PC cells were cultured on the slides overnight, fixed with 4% paraformaldehyde for 30 min, and permeabilized with 0.1% Triton-X100 (Boster Biological Technology) for 5 min. After blocking with 5% BSA for 1 h, the cells were then incubated with primary antibodies against YWHAG (1:250) and RAF1 (1:200) or p-ERK (1:100) at 4 °C overnight. CY3-conjugated goat anti-rabbit and Fluorescein isothiocyanate (FITC)-conjugated goat anti-mouse secondary antibodies were diluted 1:200 in blocking buffer and applied to cells at 37 °C for 1 h, followed by stained with DAPI for 5 min. The laser scanning confocal microscope was applied to acquire the images.

### RNA fluorescence in situ hybridization (FISH)

The specific FISH probe of CERS6-AS1 was purchased from Ribobio. PC cells were fixed with 4% paraformaldehyde at 37 °C for 30 min. Then, the cells were washed with PBS and permeabilized with 0.1% Triton-X100 for 5 min, followed incubated with the FISH probe for 2 h. Lastly, the cell nuclei were stained with DAPI for 5 min. The laser scanning confocal microscope was applied to capture the images.

### Statistical analysis

The experiments were replicated three times in the laboratory. SPSS 21.0 software and GraphPad 8.0 software were used for statistical analyses. All data are expressed as the mean ± standard deviation. Statistical analysis was performed using the Student’s *t*-test or one-way analysis of variance. The correlations between CERS6-AS1 expression and various clinicopathological features were analyzed by Fisher’s exact test. A difference was considered statistically significant if *p < 0.05, **p < 0.01, or ***p < 0.001.

## Results

### CERS6-AS1 expression is upregulated in PC and associated with a poor prognosis

**Fig. 1:**
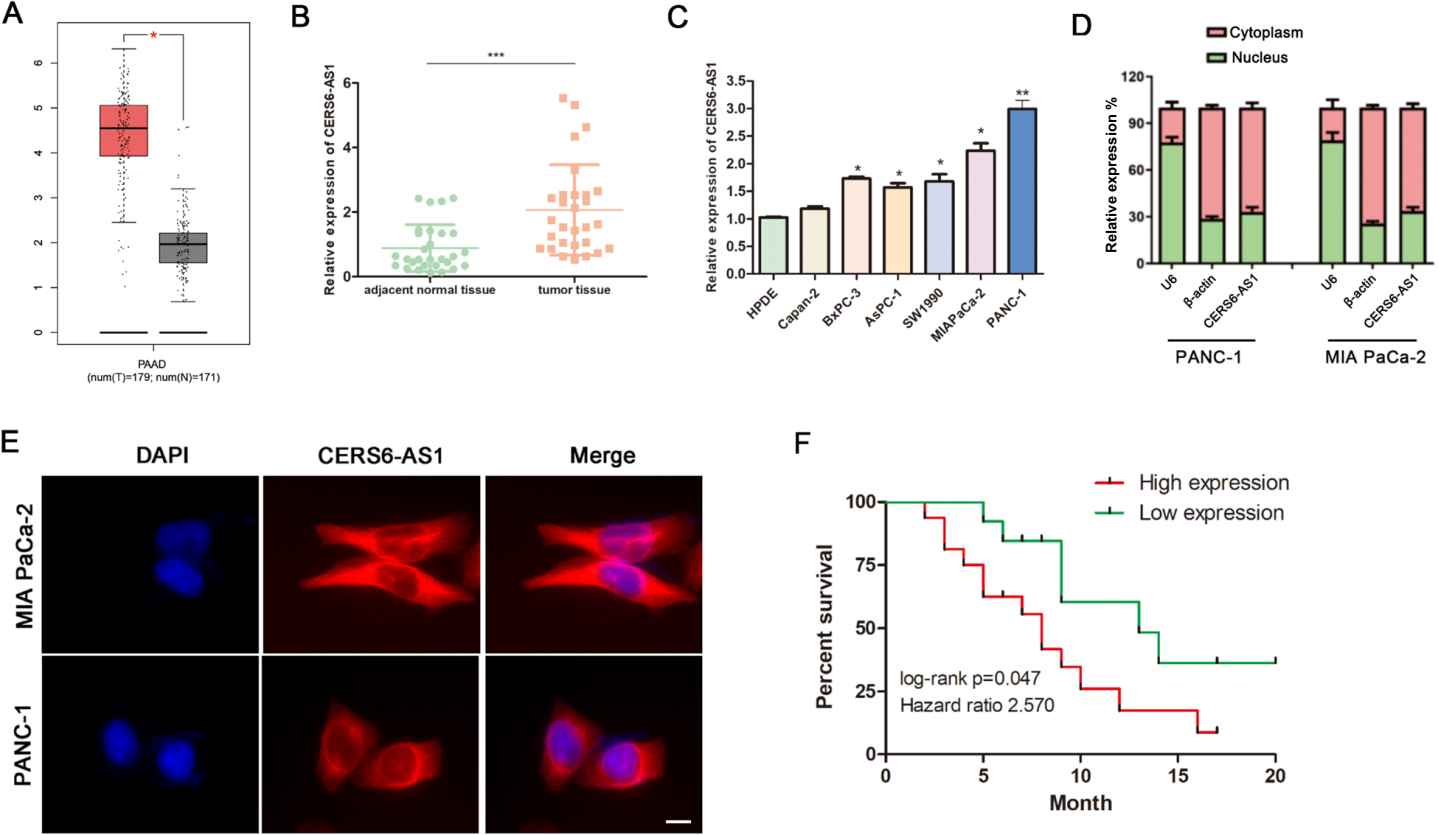
CERS6-AS1 expression is upregulated in PC and associated with poor prognosis. **A** The expression of CERS6-AS1 in PC tissues obtained from the TCGA database. **B** RT-qPCR analysis of CERS6-AS1 expression in PC tissues. **C** A differential expression pattern of CERS6-AS1 was observed in PC cell lines. **D** The relative expression of CERS6-AS1 in nucleus and cytoplasm, the U6 and β-actin were used to serve as the internal reference of nucleus and cytoplasm respectively. **E** RNA-FISH localization of CERS6-AS1 in PC cells (scale bar: 12.5 μm). **F** Survival curve of PC patients with high and low CERS6-AS1 expression. All experiments were performed three times and data were presented as mean ± SD. *p < 0.05, **p < 0.01, ***p < 0.001.

To analyze the involvement of differentially expressed lncRNAs in PC, we investigated the lncRNAs with markedly different expression via the gene expression profiling interactive analysis (GEPIA) network database (http://gepia.cancer-pku.cn/), which is based on TCGA datasets. We found that CERS6-AS1 was significantly upregulated in tumor tissues compared with normal pancreas tissues (Fig. 1A). In addition, we analyzed the GEO database (GSE63124) and found that CERS6-AS1 was highly expressed in primary tumors compared with metastases (Supplementary Fig. 1). To further confirm the expression of CERS6-AS1 in PC tissues, we measured the mRNA level of CERS6-AS1 in PC tissues and their paired adjacent non-tumor tissues by qRT-PCR, and the results suggested that CERS6-AS1 expression was significantly elevated in PC tissues (Fig. 1B). Subsequently, we detected the expression of CERS6-AS1 in different PC cell lines by qRT-PCR, and all PC cell lines showed a higher expression level than HPDE cells. The two cell lines PANC-1 and MIA PaCa-2 displayed the highest expression and were thus selected for subsequent studies (Fig. 1C). To identify the localization of CERS6-AS1 in PC cells, CERS6-AS1 RNA was isolated from the cell cytoplasm and nucleus using the Cytoplasmic and Nuclear RNA Purification Kit. PCR analysis revealed that there were more CERS6-AS1 RNA locating in the cytoplasm of PC cells (Fig. 1D). Besides, the result was further validated by FISH analysis, indicating CERS6-AS1 mainly located in the cytoplasm (Fig. 1E). Additionally, we analyzed the relationship between CERS6-AS1 expression and clinicopathologic characteristics. PC cases (*n* = 29) were divided into high and low CERS6-AS1 expression groups according to the median value. The results demonstrated that high CERS6-AS1 expression was significantly correlated with larger tumor size and lymphatic metastasis (Table 1). Moreover, Kaplan–Meier analyses revealed that increased CERS6-AS1 expression was significantly associated with poor prognosis in PC patients (Fig. 1F).

**Table 1:** General clinicopathological characteristics of patients.

Clinicopathologic feature	CERS6-AS1		*p* value
	High expression	Low expression	
All cases	16	13	
Age			
≤50	5	6	0.4657
>50	11	7	
Gender			
Male	9	5	0.4621
Female	7	8	
Diameter of tumor			
≤2	5	10	**0.0253**
>2	11	3	
TNM stage			
I/II	10	10	0.4543
III/IV	6	3	
Lymphatic metastasis			
Negative	4	9	**0.0271**
Positive	12	4	
Distant metastasis			
Negative	13	12	0.6059
Positive	3	1	
Pathological grading			
I/II	12	8	0.6882
III/IV	4	5	

The bold values mean *p* < 0.05

### CERS6-AS1 downregulation inhibits PC proliferation and invasion in vitro

**Fig. 2:**
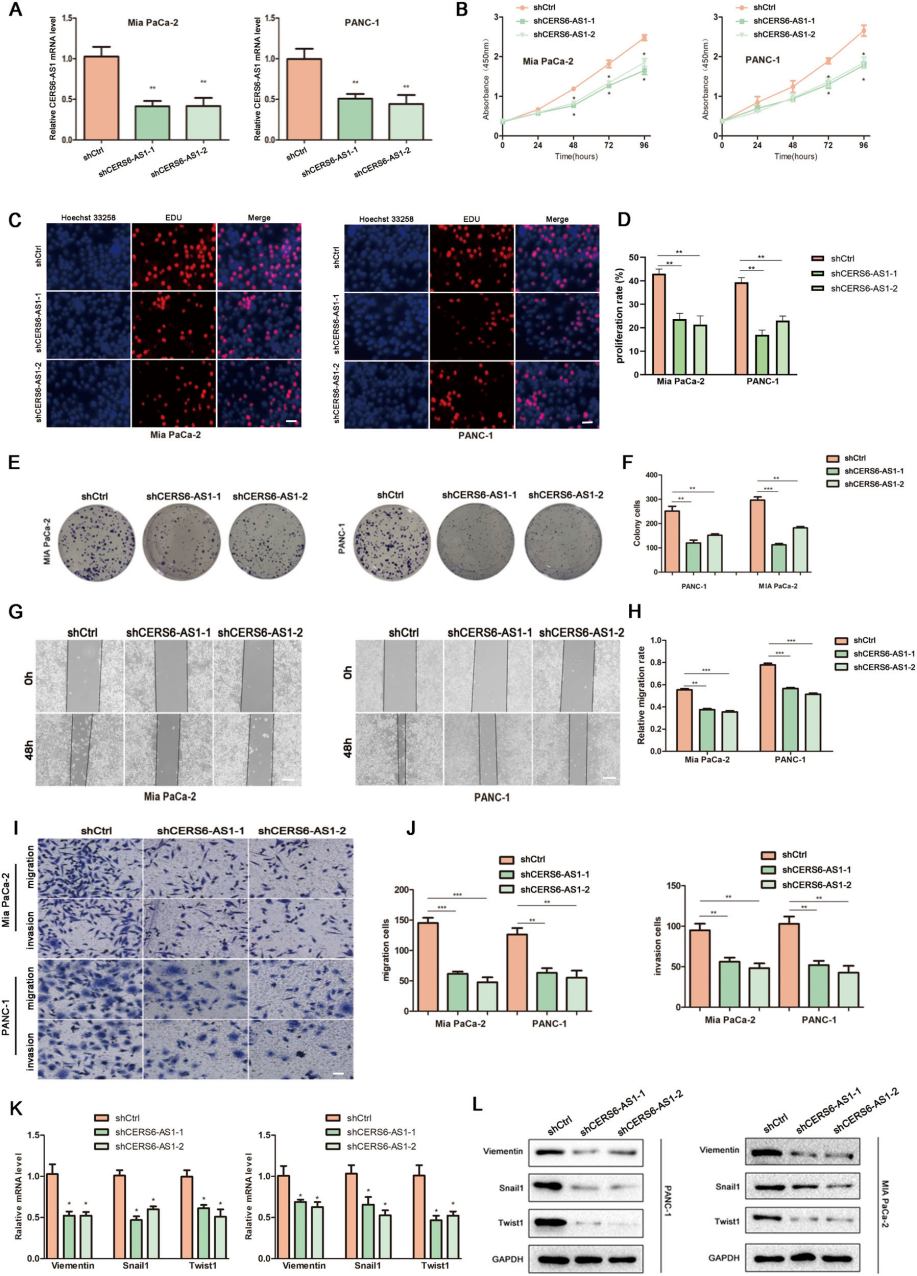
CERS6-AS1 downregulation inhibits PC proliferation and invasion in vitro. **A** The effects of transfection of lentiviral transduction in PC cell lines (MIA PaCa-2, PANC-1) were detected by PCR analysis. **B** CCK-8 assay was performed to test the cell viability and proliferation in downregulated CERS6-AS1 groups (shCERS6-AS1) and negative control group (shCtrl). **C**, **D** EDU assays were performed to measure the proliferation ability of PANC-1 and MIA PaCa-2 (scale bar: 100 μm). **E**, **F** Colony formation assay was performed to test the cell colony ability in downregulated CERS6-AS1 groups compared to shCtrl. **G**, **H** The migrated ability of MIA PaCa and PANC-1 was measured by wound healing assay (scale bar: 250 μm). **I**, **J** Transwell assay was performed the migrated and invaded abilities in PC cell lines (scale bar: 100 μm). The mRNA (**K**) and the protein (**L**) expressions of Vmentin, Twist1, and Snail1 were respectively detected in shCRES6-AS1 and shgroups by PCR analysis and western blotting. *p < 0.05, **p < 0.01, ***p < 0.001.

To investigate the effects of CERS6-AS1 expression on malignant phenotypes, we first transfected lentiviruses with silencing sequences targeting CERS6-AS1 to establish stable CERS6-AS1 knockdown cell lines (PANC-1 and MIA PaCa-2). The transfection efficiency was confirmed by qRT-PCR analyses, and the results showed that the two different silencing sequences successfully downregulated CERS6-AS1 expression (Fig. 2A). CCK-8 assays were performed to detect the cell viability, and the results indicated decreased cell viability in both knockdown cell lines (Fig. 2B). These results were validated via EDU assays, which revealed that downregulated CERS6-AS1 expression substantially suppressed PC cell proliferation (Fig. 2C, D). In addition, the colony formation assay results suggested that CERS6-AS1 downregulation significantly reduced cell colony formation compared with the negative control group (Fig. 2E, F). To evaluate the metastatic ability of PC cells in shCrtl and shCERS6-AS1 groups, wound healing assays were performed, and the results revealed that downregulated CERS6-AS1 expression inhibited cell migration (Fig. 2G, H). These results were further confirmed via transwell assays, which suggested that CERS6-AS1 downregulation clearly impaired the migration and invasion of MIA PaCa-2 and PANC-1 cells (Fig. 2I, J). Subsequently, we detected the relative indexes of EMT by PCR and western blot, and the results indicated that the mRNA and protein levels of Vimentin, Snail1, and Twist1 were significantly decreased in the shCERS6-AS1 groups compared with the shCtrl groups (Fig. 2K, L). These data demonstrated that CERS6-AS1 positively regulated the proliferation and invasion of PC cells.

### CERS6-AS1 downregulation suppresses PC growth and invasion in vivo

**Fig. 3:**
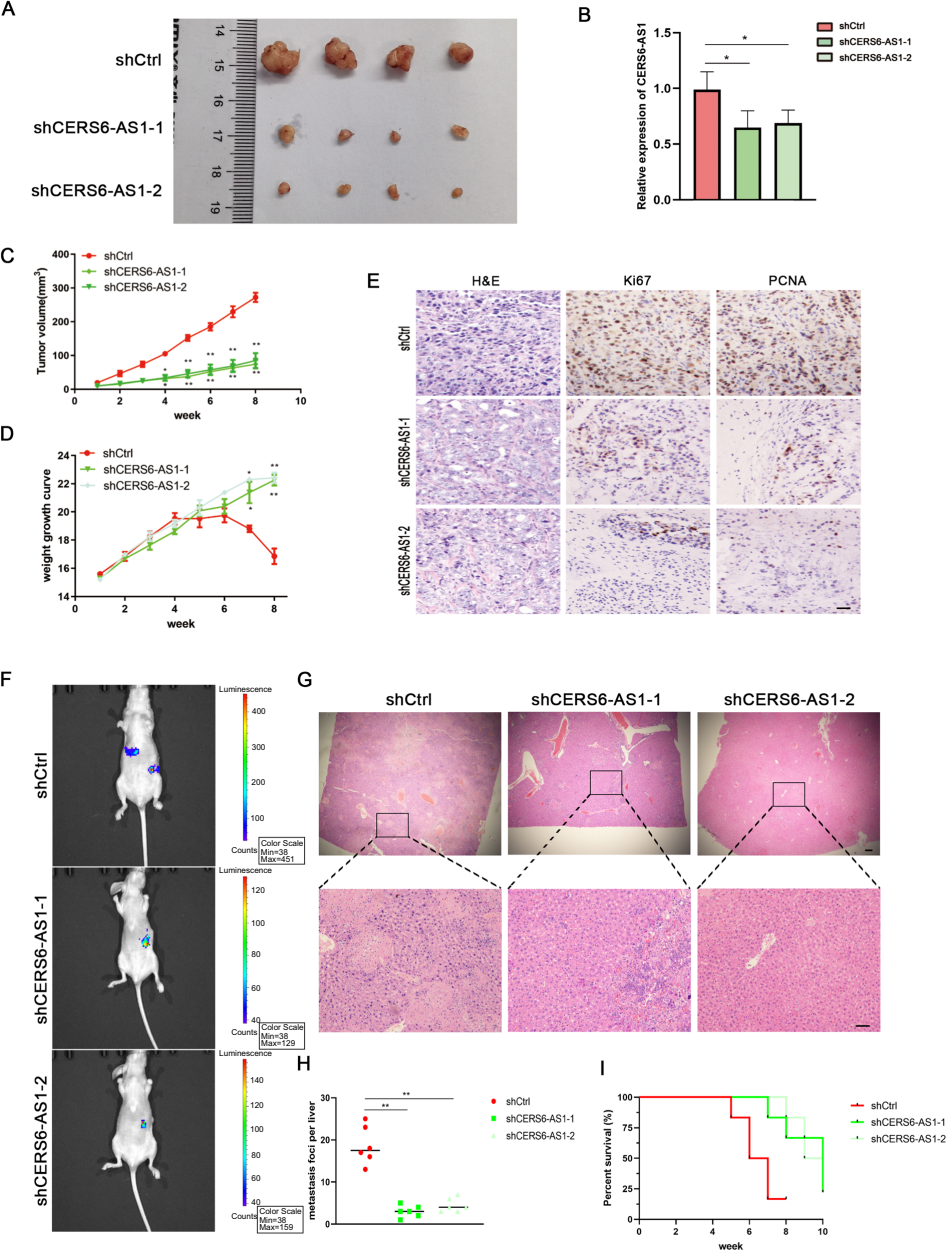
CERS6-AS1 downregulation inhibits PC proliferation and invasion in vivo. **A** Tumor photographs of the subcutaneous xenografts in shCtrl and shCERS6-AS1 groups, *n* = 4, cell line: PANC-1. **B** The relative RNA expression of CERS6-AS1 in subcutaneous tumors was detected by PCR. **C** Tumor volume of the subcutaneous xenografts in shCtrl and shCERS6-AS1. **D** Weight change curve. **E** IHC staining for CERS6-AS1 and representative images of three pairs of subcutaneous xenograft tissue (scale bar: 100 μm). **F** The PANC-1 cells were conducted the metastasis model for 10 weeks, and the in vitro imaging indicated the metastasis loci, *n* = 6 (Colors indicate metastasis existing, Red indicates high enrichment, blue indicates low enrichment). **G** Serial sections of whole liver were H&E stained (scale bar: 100 μm). **H** The survival curve showed the prognosis of mice in shCtrl and shCERS6-AS1 groups. **I** Liver micrometastases were counted. *p < 0.05, **p < 0.01.

To further verify the oncogenic function of CERS6-AS1 in PC cells in vivo, we established a subcutaneous implantation model and a metastasis model. CERS6-AS1 knockdown in PANC-1 cells resulted in a decreased tumor size in shCRES6-AS1 groups compared with the shCtrl group (Fig. 3A). Then, we extracted total RNA from tumors in the shCRES6-AS1 groups and shCtrl group and detected the mRNA level of CERS6-AS1 by qRT-PCR. The results revealed that CERS6-AS1 expression was downregulated in the shCRES6-AS1 groups (Fig. 3B). Meanwhile, CERS6-AS1-deficient tumors showed a smaller tumor volume than those from the shCtrl group (Fig. 3C). Additionally, the nude mice in the shCtrl group started to present weight loss after 4 weeks, probably mice in negative control groups were more trend to cachexia than the nude mice in the CERS6-AS1 inhibition groups (Fig. 3D). IHC analysis of the tumors suggested that Ki67 and PCNA, two proliferation indexes, were more highly expressed in the shCtrl group than in the CERS6-AS1 inhibition groups (Fig. 3E). In addition, the in vitro imaging results suggested that the bioluminescence was mainly concentrated in the spleen in the shCRES6-AS1 groups, whereas the control group showed significant bioluminescence in the liver, suggesting increased metastasis in the control group (Fig. 3F). The liver tissue sections were observed under a microscope, which revealed that CERS6-AS1 knockdown resulted in significantly fewer liver metastases (Fig. 3G, H). Interestingly, CERS6-AS1 downregulation was correlated with a significantly improved prognosis (Fig. 3I).

### CERS6-AS1 functions as a sponge for miR-217 and miR-217 inhibits PC proliferation and invasion

**Fig. 4:**
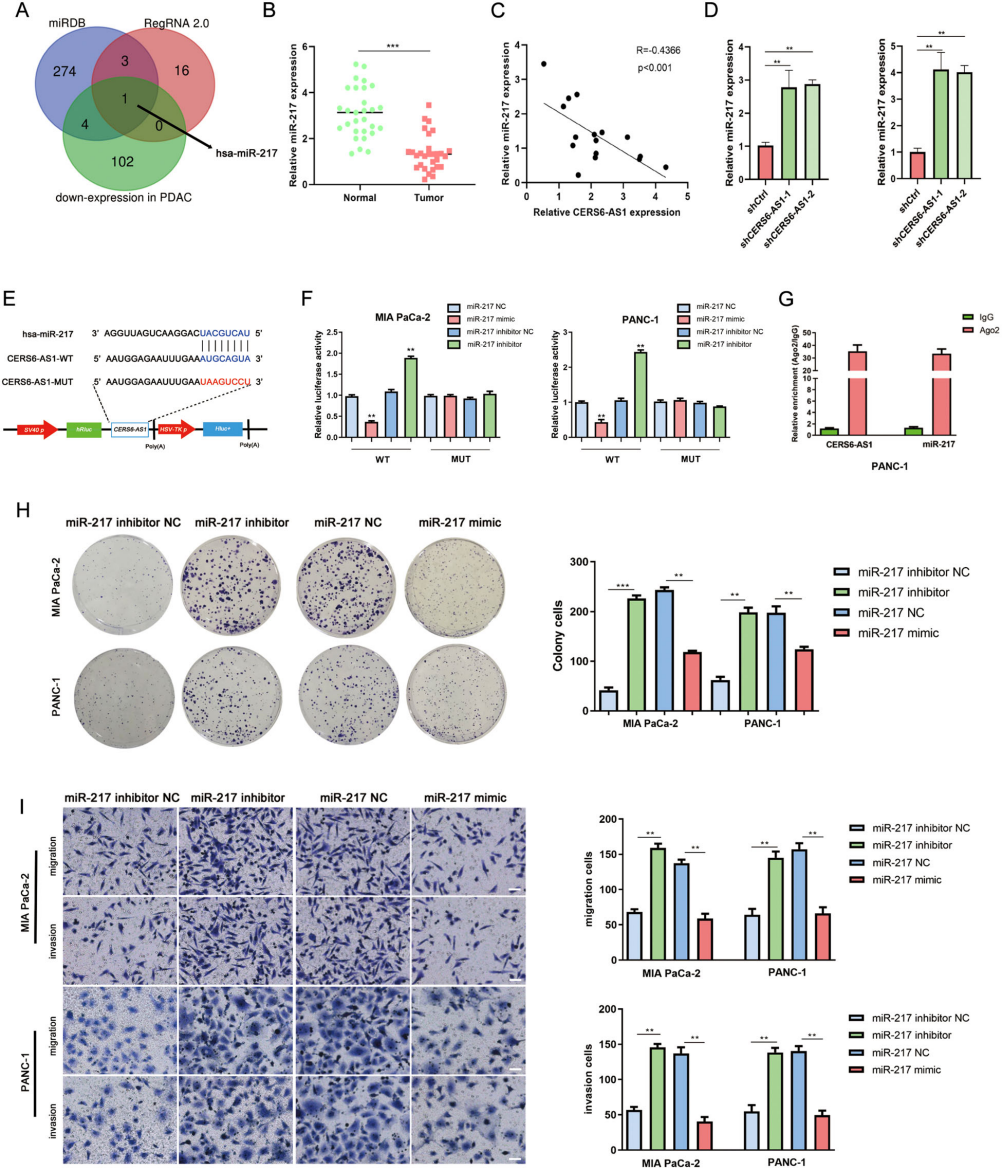
CERS6-AS1 behaves like a sponge for miR-217 and miR-217 inhibits PC proliferation and invasion. **A** miR-217 was identified as the target of CERS6-AS1 by Venn analysis. **B** RT-qPCR analysis of miR-217 expression in PC tissues. **C** Pearson correlation analysis was used to investigate the correlation between CERS6-AS1 and miR-217. **D** miR-217 expression in shCtrl and shCERS6-AS1 groups. **E** The prediction binding site between miR-217 and CERS6-AS1. **F** Luciferase assay was applied to investigate the direct interaction between CERS6 and miR-217. **G** RIP analysis demonstrated the co-interaction of CERS6-AS1 and miR-217 with Ago2. **H** Functional experiments were performed to verify the effect of miR-335-5p mimic or inhibitor on proliferation in MIA PaCa-2 and PANC-1. **I** Functional experiments were performed to verify the effect of miR-217 mimic or inhibitor on migration and invasion in MIA PaCa-2 and PANC-1 (scale bar: 100 μm). **p < 0.01, ***p < 0.001.

To identify the candidate miRNAs of CERS6-AS1, we first analyzed the GEO database (GSE85589) and found that 107 miRNAs were downregulated in pancreatic ductal adenocarcinoma after removing the duplications. Then, the sequence of CERS6-AS1 was entered into miRDB (http://www.mirdb.org/) and RegRNA 2.0 (http://regrna2.mbc.nctu.edu.tw/) to predict the potential target miRNAs of CERS6-AS1. Finally, Venn analysis was performed to obtain the intersection of the above results, and miR-217 was identified as the only candidate miRNA of CERS6-AS1 (Fig. 4A). Subsequently, the expression of miR-217 in PC tissues and their paired normal tissues was detected by qRT-PCR, and the results verified that miR-217 was downregulated in PC tissues (Fig. 4B). There was a negative correlation between CERS6-AS1 and miR-217 expression via Pearson correlation curve analysis (Fig. 4C). the qRT-PCR analysis uncovered that miR-217 expression was enhanced in the shCERS6-AS1 groups (Fig. 4D). The predicted potential interaction site for miR-217 and CERS6-AS1 was displayed in Fig. 4E. The results of Luciferase reporter assays confirmed this binding site. The wild-type CERS6-AS1 reporter revealed lower luciferase activity in the miR-217 upregulation group and higher luciferase activity in the miR-217 downregulation group, whereas the luciferase activity in the mutant CERS6-AS1 reporter remained unchanged in the miR-217 perturbation groups (Fig. 4F). The direct interaction between CERS6-AS1 and miR-217 was further verified by RIP assays, which were enriched in the Ago2 complex (Fig. 4G). In addition, cell functional experiments indicated that upregulated expression of miR-217 obviously suppressed the growth, migration, and invasion of PC cells. Conversely, miR-217 inhibition clearly promoted cell growth and invasion (Fig. 4H, I).

### CERS6-AS1 promotes cell growth and invasion by targeting the miR-217/YWHAG axis

**Fig. 5:**
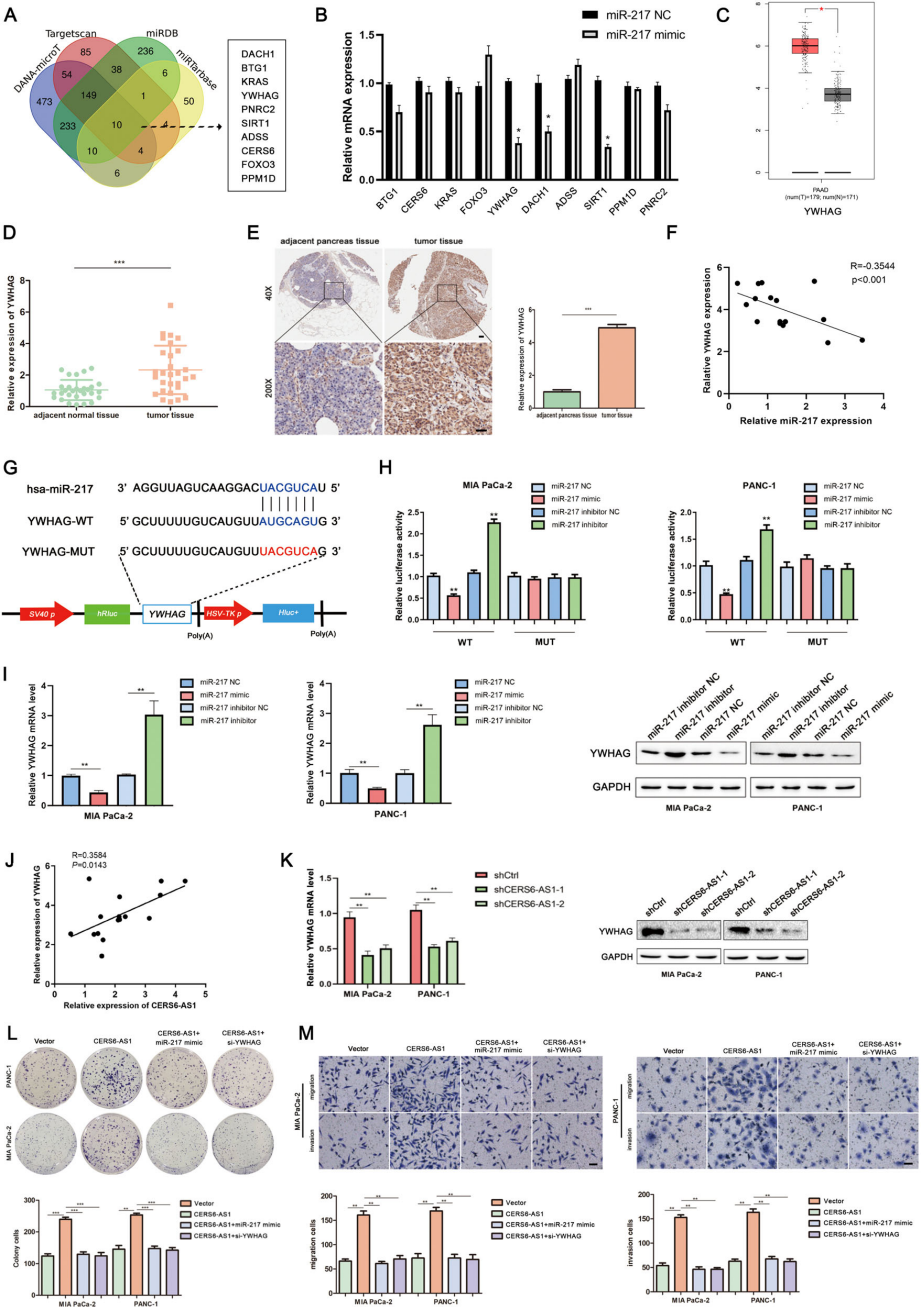
CERS6-AS1 promotes cell growth and invasion by targeting the miR-217/YWHAG axis. **A** Ten candidate genes were identified as the potential targets of miR-217 by Venn analysis. **B** Ten candidate genes were selected for subsequent PCR validation. **C** The relative YWHAG expression in PC tissues based on the TCGA database. **D** The relative YWHAG expression in our collected PC tissues. **E** The IHC assay was performed to further confirmed YWHAG expression in PC specimens (scale bar: 50 μm). **F** Pearson correlation analysis was used to investigate the correlation between YWHAG and miR-217. **G** The prediction binding site of miR-217 in YWHAG mRNA 3′UTR. **H** Luciferase assay was applied to investigate the direct interaction between YWHAG and miR-217. **I** The mRNA and protein expression of YWHAG were detected by PCR after transfecting miR-217 mimics or inhibitors in PC cells. **J** Pearson correlation analysis was used to investigate the correlation between YWHAG and CERS6-AS1. **K** The mRNA and protein expression of YWHAG were detected in shCtrl and shCERS6-AS1 groups by PCR analysis and western blotting. **L**, **M** Functional rescue experiments were performed to verify the effect of miR-217 overexpression or YWHAG knockdown on migration and proliferation in the CERS6-AS1 overexpression group (scale bar: 100 μm). *p < 0.05, **p < 0.01, ***p < 0.001.

To identify the candidate target genes of miR-217, Venn analysis was performed to obtain the intersection of the candidate genes from four bioinformatics sites, including miRTarbase (http://mirtarbase.cuhk.edu.cn/php/index.php), miRDB (http://www.mirdb.org/), DANA-microT(http://diana.imis.athena-innovation.gr/DianaTools/index.php?r=MicroT_CDS/index) and targets can (http://www.targetscan.org/vert_72/) (Fig. 5A). Ten candidate genes were selected for subsequent PCR validation, and the results indicated that *YWHAG, DACH1*, and *SIRT1* were downregulated in the miR-217 overexpression group, whereas no significant difference in the remaining genes was observed when PANC-1 cells were transfected with miR-217 mimics (Fig. 5B). To further screen the candidate genes, we investigated the expression of YWHAG, DACH1, and SIRT1 using data from the TCGA database through GEPAI. The results suggested that YWHAG expression was significantly elevated in PC tissues compared with normal tissues (Fig. 5C). However, the expression of DACH1 and SIRT1 showed no significant difference (Supplementary Fig. 2A). Kaplan–Meier analysis of overall survival and disease-free survival using patient follow-up information from the Kaplan–Meier Plotter based on the TCGA database (http://kmplot.com/analysis/index.php?p=background) (Supplementary Fig. 2B) indicated high YWHAG expression was positively correlated with poor overall survival. Subsequently, we measured the mRNA level of YWHAG in PC tissues and cell lines via PCR. The expression of YWHAG in PC tissues was consistent with the above results based on the TCGA database (Fig. 5D). However, analysis of the YWHAG expression profile in cell lines displayed that YWHAG mRNA and protein expression levels were mainly elevated in PC cell lines except for Capan-2 and BxPC-3 cells (Supplementary Fig. 2D, E). IHC analysis of PC specimens further confirmed that YWHAG was highly expressed (Fig. 5E). The correlation between YWHAG and miR-217 expression was negative using Pearson correlation curve analysis (Fig. 5F). Figure 5G showed the predicted potential interaction site between YWHAG and miR-217. And the potential interaction site was further confirmed via Luciferase reporter assays. The wild-type YWHAG reporter suggested that the luciferase activity was low in the miR-217 upregulation group and high in the miR-217 silencing group, whereas the mutant-type YWHAG reporter remained unchanged in the miR-217 perturbation groups (Fig. 5H). Meanwhile, PCR analysis, western blot, and Pearson correlation curve analysis confirmed that the correlation between YWHAG and miR-217 was negative and the expression of YWHAG was negatively regulated by miR-217 (Fig. 5I, J). However, a positive correlation between YWHAG and CERS6-AS1 expression was verified via Pearson correlation curve analysis (Fig. 5J). PCR and western blot analysis showed similar results (Fig. 5K). The rescue experiments based on cell function demonstrated that elevated miR-217 or silenced YWHAG could partly impair the ability of overexpressed CERS6-AS1 to enhance cell proliferation and metastasis (Fig. 5L, M).

### YWHAG binds to RAF1 and activates ERK signaling

**Fig. 6:**
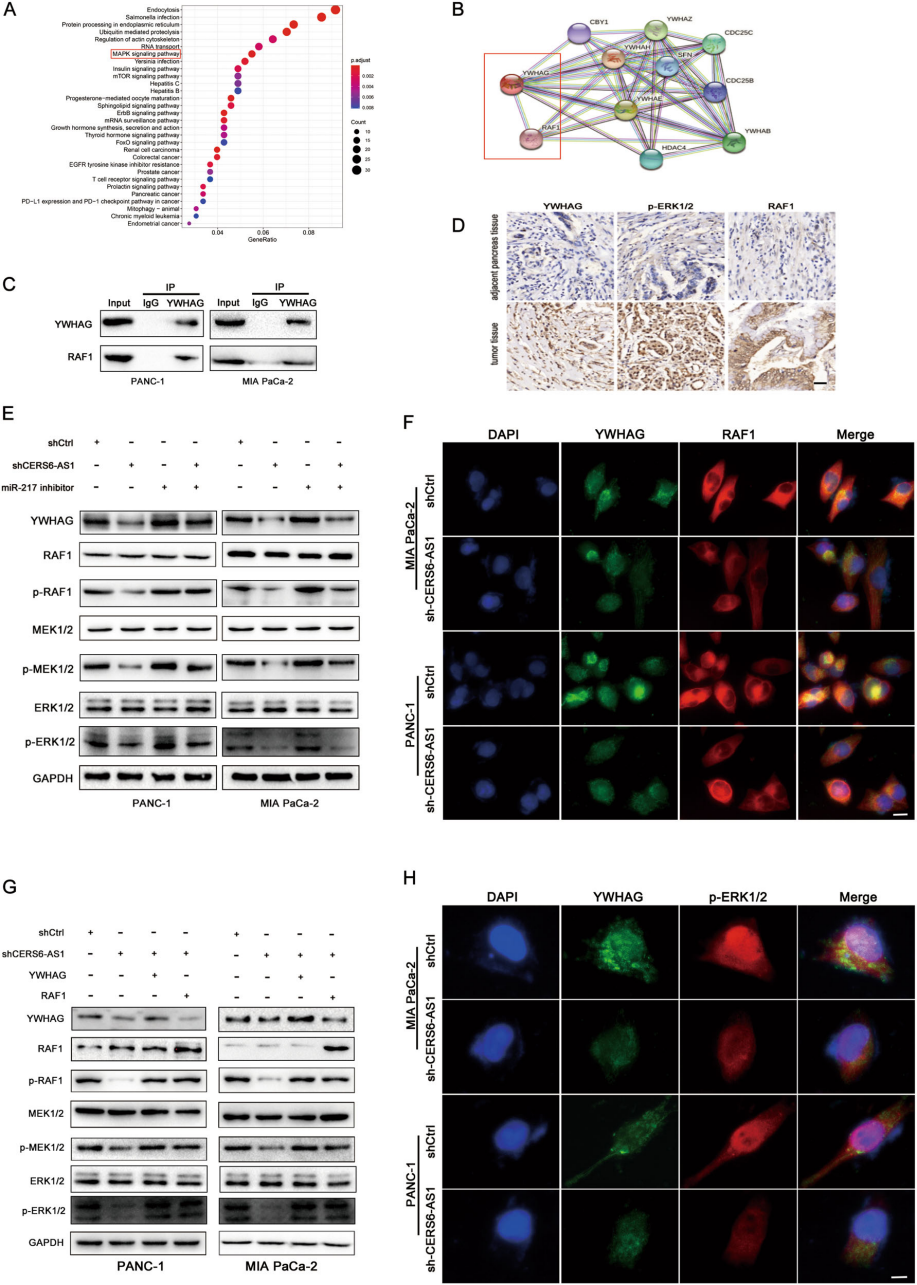
YWHAG binds to RAF1 and activates ERK signaling. **A** KEGG pathway enrichment analysis was performed to predict the downstream signal pathway of YWHAG. **B** Co-expression genes analysis was performed to predict the potential genes existing interaction with YWHAG via the STRING 11.0 network database. **C** The interaction between YWHAG and RAF1 was validated by Co-immunoprecipitation. **D** The correlation among YWHAG, RAF1, and p-ERK was validated by IHC from PC specimens (scale bar: 50 μm). **E** the protein expression changes of YWHAG, RAF1, MEK1/2, ERK1/2 and phosphorylated-RAF1, phosphorylated-MEK1/2, phosphorylated-ERK1/2 in shCERS6-AS1 and miR-217 inhibitor compared to shCtrl and shCERS6-AS1 + miR-217 inhibitor groups. **F** Immunofluorescence assays were performed to further validate the interaction between RAF1 and YWHAG (scale bar: 12.5 μm). **G** the protein expression changes of YWHAG, RAF1, MEK1/2, ERK1/2 and phosphorylated-RAF1, phosphorylated-MEK1/2, phosphorylated-ERK1/2 in shCtrl, shCERS6-AS1, shCERS6-AS1 + YWHAG and shCERS6-AS1 + RAF1 groups, respectively. **H** Immunofluorescence assays were used to determine whether the expression of YWHAG affected the localization of p-ERK (scale bar: 12.5 μm).

**Fig. 7:**
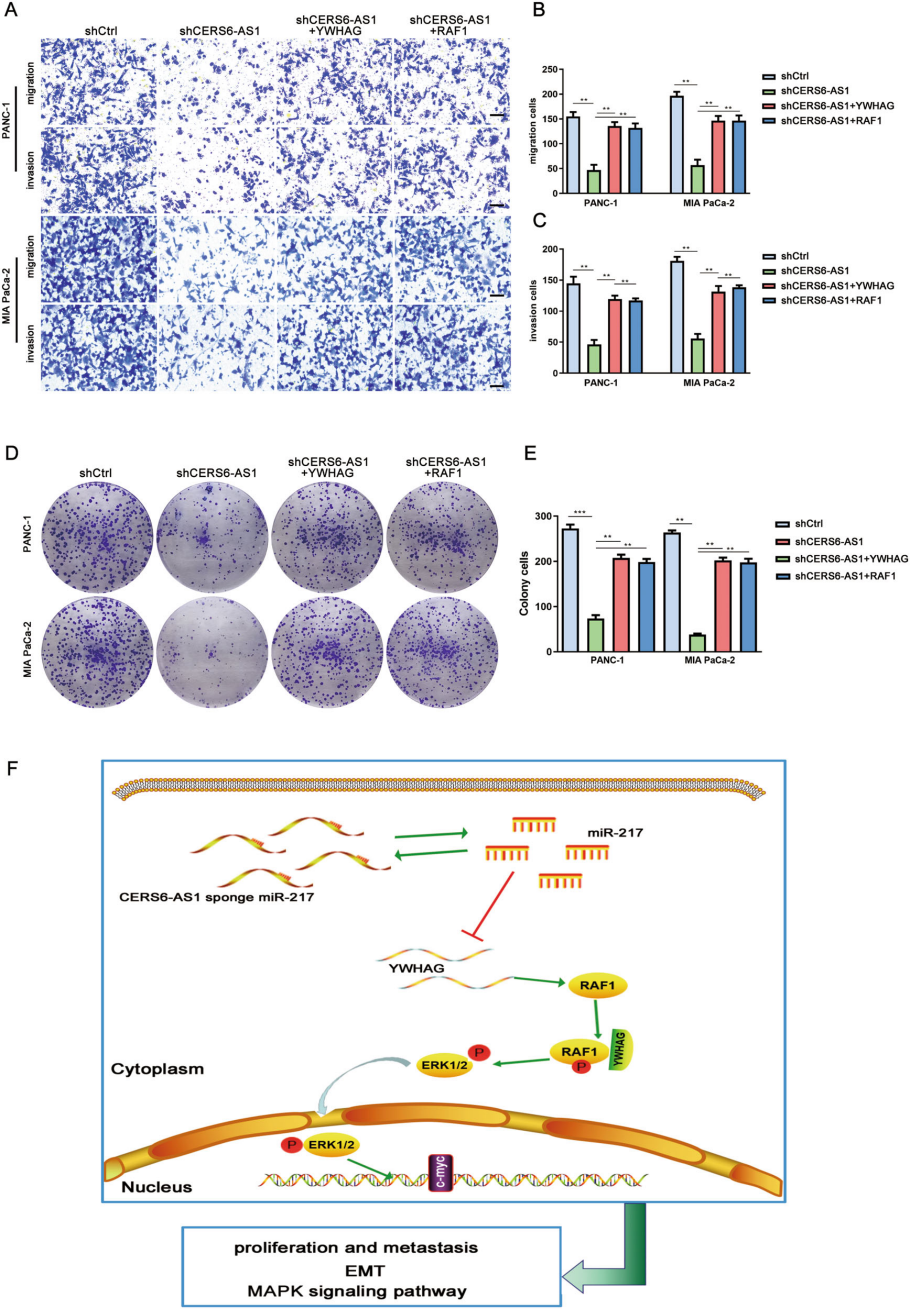
CERS6-AS1 promotes PC cell proliferation and metastasis by activating ERK signaling via miR-217/YWHAG/RAF1 axis. **A**–**C** Functional rescue experiments were performed to validated migration and invasion in shCERS6-AS1 + YWHAG and shCERS6-AS1 + RAF1 groups compared to shCRES6-AS1 group (scale bar: 100 μm). **D**, **E** Functional rescue experiments were performed to validated proliferation in shCERS6-AS1 + YWHAG and shCERS6-AS1 + RAF1 groups compared to the shCRES6-AS1 group. **F** The schematic diagram is used to elucidate the underlying molecular mechanism that CERS6-AS1 promotes PC metastasis and proliferation and indirectly upregulates YWHAG and phosphorylates of RAF1 to activate ERK signaling by decoying miR-217.

KEGG pathway analysis of the signaling pathways downstream from YWHAG revealed that the MAPK pathway was significantly enriched (Fig. 6A). Gene co-expression analysis using the STRING 11.0 network database (https://string-db.org/) suggested a potential interaction between YWHAG and RAF1 (Fig. 6B). Co-immunoprecipitation (Co-IP) and immunofluorescence assays were performed to further validate this hypothesis. The results demonstrated that RAF1 protein was pulled down by YWHAG, suggesting an interaction between RAF1 and YWHAG (Fig. 6C). In addition, both proteins were co-localized in the cytoplasm of PC cells, and the amount of co-localization was regulated by the expression of CERS6-AS1 (Fig. 6F). Meanwhile, fluorescence quantification analysis was performed using Image J software, and the results suggested that silenced CERS6-AS1 induced the decreasing number of interactions between YWHAG and RAF1 (Supplementary Fig. 3). Quantification of colocalization between YWHAG and RAF1 staining expressed as overlap coefficients in supplementary Figure 3. The higher score of overlap coefficient represents the better colocalization between YWHAG and RAF1. As our data depicted, the knockdown of CERS6-AS1 significantly attenuated colocalization between YWHAG and RAF1 (Supplementary Fig. 3). IHC analysis of PC specimens suggested a positive correlation among the expression of YWHAG, RAF1, and p-ERK1/2 (Fig. 6D). Western blot analyses suggested that CERS6-AS1 silencing could downregulate YWHAG expression without changing the total expression of RAF1. However, RAF1 phosphorylation levels were reduced following CERS6-AS1 downregulation, revealing that YWHAG could enhance RAF1-mediated ERK signaling activation. Interestingly, suppression of miR-217 expression could partly rescue the decreased phosphorylation induced by suppressing CERS6-AS1 expression (Fig. 6E). In addition, RAF1 or YWHAG overexpression could also partly rescue the reduced phosphorylation induced by suppressing CERS6-AS1 expression (Fig. 6G). IF results indicated that p-ERK1/2 was more likely to be translocated from the cytoplasm to the nucleus to mediate transcriptional regulation in the control groups compared with the shCERS6-AS1 groups, further suggesting that CERS6-AS1 could activate ERK signaling via the miR-217/YWHAG/RAF1 axis (Fig. 6H). Unsurprisingly, the upregulated expression of YWHAG or RAF1 could partly rescue the inhibitory function on growth and migration induced by CERS6-AS1 knockdown (Fig. 7A–E). This study demonstrates that CERS6-AS1 exerts a vital role in accelerating PC progression by upregulating YWHAG and phosphorylating RAF1 to activate ERK signaling (Fig. 7F). These findings highlight the CERS6-AS1/miR-217/YWHAG axis as a potential target for the early diagnosis and personalized treatment of PC.

## Discussion

Recent evidence indicates that lncRNAs are involved in regulating malignant phenotypes, such as enhanced cell proliferation, drug resistance, distant metastasis, and vascular invasion [12]. Interestingly, several lncRNAs are dysregulated and serve as tumor promoters or suppressors in cancer progression. For example, LINC01057 and IKKα interactions contribute to glioblastoma mesenchymal differentiation by maintaining IKKα nuclear localization and activating NF-κB signaling [13]. However, LINC00657 suppresses cervical cancer malignant phenotype by competitively interacting with miR-20a-5p [7]. In PC, LINC00337 acts as an E2F1 coactivator to activate the expression of target proteins and promote cell growth and cell cycle transition [14]. Methylation-mediated LINC00261 functions as a suppressor to inhibit PC cell proliferation, migration, and metastasis through interacting with the bromodomain of p300/CBP to repress c-Myc transcription [15]. In this study, we found that the CERS6-AS1 expression level was elevated in PC tissues and positively associated with larger tumor size, lymphatic node metastasis, and poor overall survival. The above findings indicated that CERS6-AS1 might participate in PC progression.

CERS6-AS1 was first reported in breast cancer, and high CERS6-AS1 expression was positively correlated with malignant phenotypes. Mechanistically, CERS6-AS1 exerted oncogenic functions by modulating the stability of CERS6 mRNA via binding to RNA-binding proteins (IGF2BP3) [16]. Additionally, CERS6-AS1 functioned as a ceRNA to regulate miR-125a-5p, upregulate BAP1 expression and promote breast cancer cell growth [8]. The study by Zhang et al suggested that CERS6-AS1 was significantly overexpressed in hepatocellular carcinoma, and patients with CERS6-AS1 upregulation exhibited a poor prognosis [9]. CERS6-AS1 was also identified as a novel potential Alzheimer’s disease biomarker and was markedly correlated with neuronal projection development and morphogenesis [17]. In this study, we confirmed that CERS6-AS1 was mainly localized in the cytoplasm of PC cells, which was the basis of its function as a ceRNA. Bioinformatics analysis indicated that CERS6-AS1 might bind to miR-217. miR-217 participates in the regulation of various cancers, such as prostate cancer [18], lung cancer [19], thyroid cancer [20], PC [21], and others. Subsequently, we confirmed the interaction between miR-217 and CERS6-AS1 through luciferase reporter and RIP assays. Then, we determined that CERS6-AS1 promoted PC cell proliferation, migration, and invasion by sponging miR-217 to regulate the expression of YWHAG.

YWHAG is also named 14-3-3γ and is a member of the 14-3-3 family of acidic proteins involved in regulating the functions of serine/threonine-phosphorylated proteins [22]. Extensive evidence suggests that YWHAG functions as a scaffold in multiprotein complexes and participates in the regulation of tumorigenesis and progression, including cell cycle transformation and cell growth [23]. The expression of YWHAG was remarkably overexpressed in gastric cancer, and YWHAG upregulation indicated an unfavorable prognosis. YWHAG promoted gastric cancer proliferation and metastasis and inhibited cell apoptosis by activating Ras signaling to enhance Cyclin D1 and inhibit p21 and p27, which are key proteins in G1 of the cell cycle [24]. In addition, Plk1 phosphorylation at Ser99 promoted YWHAG binding to Plk1, and YWHAG specifically enhanced Plk1 activity via a mechanism independent from Plk1-Thr210 phosphorylation in mitosis, resulting in metaphase-anaphase transition [25]. The interaction between ANGPTL4 and YWHAG was positively associated with EMT, and YWHAG was necessary for ANGPTL4 to coordinate the energy demand during EMT [26]. Knockdown of YWHAG suppressed EMT and reduced the metastasis of non-small cell lung cancer [27]. Our data suggested that CERS6-AS1 promoted YWHAG overexpression by sponging miR-217. YWHAG could bind to RAF1 and increase its phosphorylation levels to activate ERK signaling. The activation of ERK signaling participates in multiple biological processes in PC, including cell proliferation and migration [28]. Supporting this data, the binding of YWHAG to RAF1 occurred in the cytoplasm. In addition, the suppression of CERS6-AS1 expression decreased the protein levels of YWHAG, p-RAF1, p-MEK1/2, and p-ERK1/2, and these effects were reversed by upregulating YWHAG in the shCERS6-AS1 groups. Furthermore, CERS6-AS1 promoted p-ERK translocation from the cytoplasm to the nucleus, suggesting that CERS6-AS1 specifically activated ERK signaling based on the binding of YWHAG to RAF1. Mechanistically, CERS6-AS1 promoted PC progression by competitively binding to miR-217 to upregulate YWHAG and promote the phosphorylation of RAF1, thereby activating ERK signaling.

## Conclusions

In summary, our study provided evidence that CERS6-AS1 was overexpressed in PC tissues and cells, and its levels were directly associated with lymph node metastasis, larger tumor size, and poor overall survival. CERS6-AS1 sponged miR-217 and indirectly upregulated the expression of YWHAG, which interacted with and promoted the phosphorylation of RAF1, leading to the activation of ERK signaling. In short, the CERS6-AS1/miR-217/YWHAG/RAF1 signaling axis might provide a biomarker for specific diagnoses or a target for individualized treatment.

## Supplementary information

Supplemental Figure and Table legends

supplemental Table 1

supplemental Figure 1

supplemental Figure 2

supplemental Figure 3

## Data Availability

All data generated and analysed during this study are included in this published article are available on request.
