# Supplementary material for: LncRNA CERS6-AS1 promotes proliferation and metastasis through the upregulation of YWHAG and activation of ERK signaling in pancreatic cancer
Source: Cell Death Dis. 2021 Jun 24;12(7):648. doi: 10.1038/s41419-021-03921-3 (PMC8225895; doi:10.1038/s41419-021-03921-3)
Supplement: Supplementary file 1 — Supplemental Figure and Table legends [file 41419_2021_3921_MOESM1_ESM.docx]

**Supplemental Figure and Table legends**

**Supplemental Table 1. The primer sequences in this study.**

**Supplemental Figure 1. CERS6-AS1 was highly expressed in PC tissues.** The expression level of LINC00941 in GEO database (GSE63124)

**Supplemental Figure 2. The expression of YWHAG was upregulated in PC tissues and cells.** (a) the expression of DACH1 and SIRT1 in GEPIA database based on TCGA database. (b) The overall survival months in patients with PC obtained from TCGA database. (c) The disease free survival months in patients with PC obtained from TCGA database. (d) The mRNA expression levels of YWHAG in PC cell lines were analyzed by PCR. (e) The protein expression levels of YWHAG in PC cell lines were analyzed by western blotting.

**Supplemental Figure 3.** **Fluorescence quantification analysis was performed by image J software.** lmageJ was used to detect the fluorescence colocalization, the detection area contains:(1) red fluorescence; (2) green flurorescene; (3)merge. Merge is the result of the colocalizetion of YWHAG and RAF1. The overlap coefficient was showed on the third graph of each line and the last graph of each line implicated the visualized coffecient of green and red fluoresence channel.
