# Supplementary material for: LncRNA CERS6-AS1 promotes proliferation and metastasis through the upregulation of YWHAG and activation of ERK signaling in pancreatic cancer
Source: Cell Death Dis. 2021 Jun 24;12(7):648. doi: 10.1038/s41419-021-03921-3 (PMC8225895; doi:10.1038/s41419-021-03921-3)
Supplement: Supplementary file 2 — supplemental Table 1 [file 41419_2021_3921_MOESM2_ESM.docx]

| Gene | Sequence | |
| --- | --- | --- |
| GAPDH | Forward | 5′-GGAGCGAGATCCCTCCAAAAT-3′ |
|  | Reverse | 5′-GGCTGTTGTCATACTTCTCATGG-3′ |
| U6 | Forward | 5′-TGCGGGTGCTCGCTTCGGC-3′ |
|  | Reverse | 5′-CCAGTGCAGGGTCCGAGGT-3′ |
| CERS6-AS1 | Forward | 5′-GCAGCCCAGCAGAAGTAGGA-3′ |
|  | Reverse | 5′-GAGCATAGGGAAGCAACTCTCAG-3′ |
| miR-217 | Forward | 5′-CGCGTACTGCATCAGGAACTG-3′ |
|  | Reverse | 5′-AGTGCAGGGTCCGAGGTATT-3′ |
| YWHAG | Forward | 5′-CCTGGCTCTTAACTACTCCGT-3′ |
|  | Reverse | 5′-GTCCTCGTTGAGGGTGTCAAG-3′ |
