# Supplementary figures and images for: LncRNA CERS6-AS1 promotes proliferation and metastasis through the upregulation of YWHAG and activation of ERK signaling in pancreatic cancer
Source: Cell Death Dis. 2021 Jun 24;12(7):648. doi: 10.1038/s41419-021-03921-3 (PMC8225895; doi:10.1038/s41419-021-03921-3)

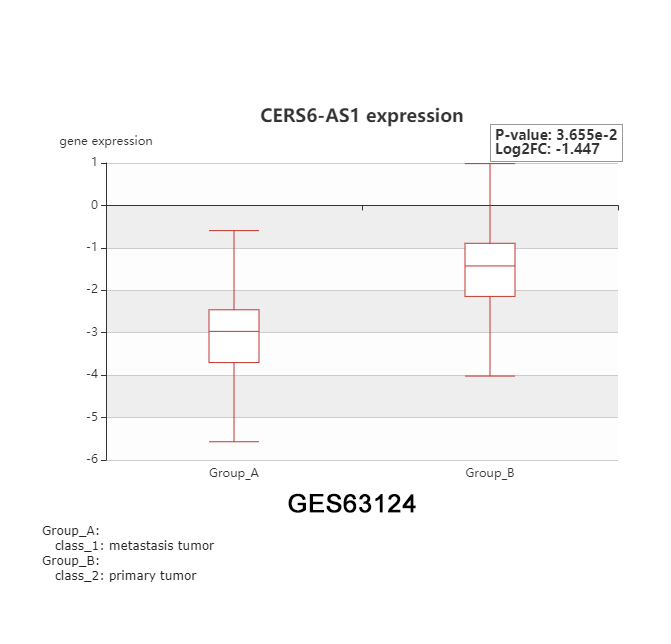

Supplement: Supplementary file 3 — supplemental Figure 1 [file 41419_2021_3921_MOESM3_ESM.tif]

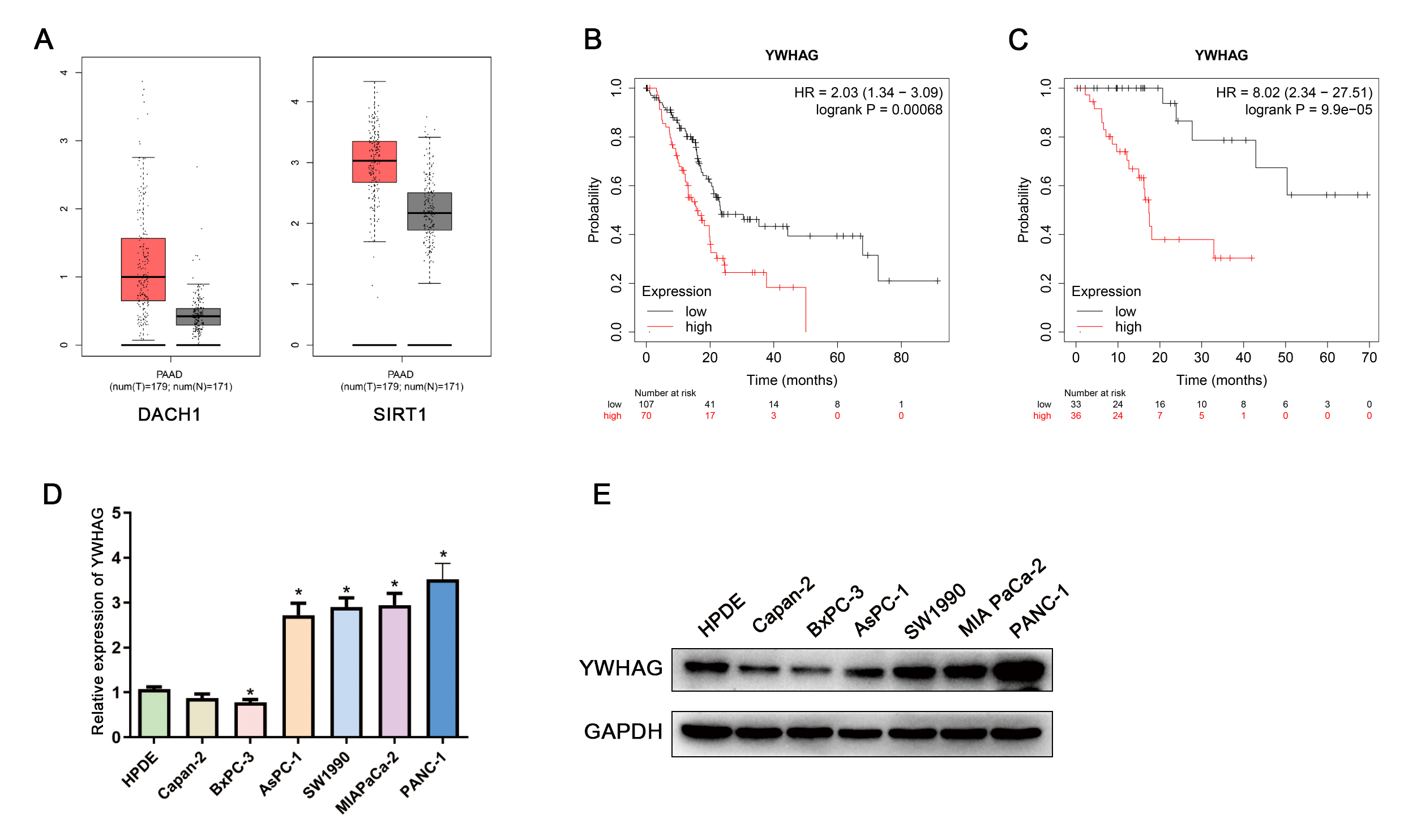

Supplement: Supplementary file 4 — supplemental Figure 2 [file 41419_2021_3921_MOESM4_ESM.tif]

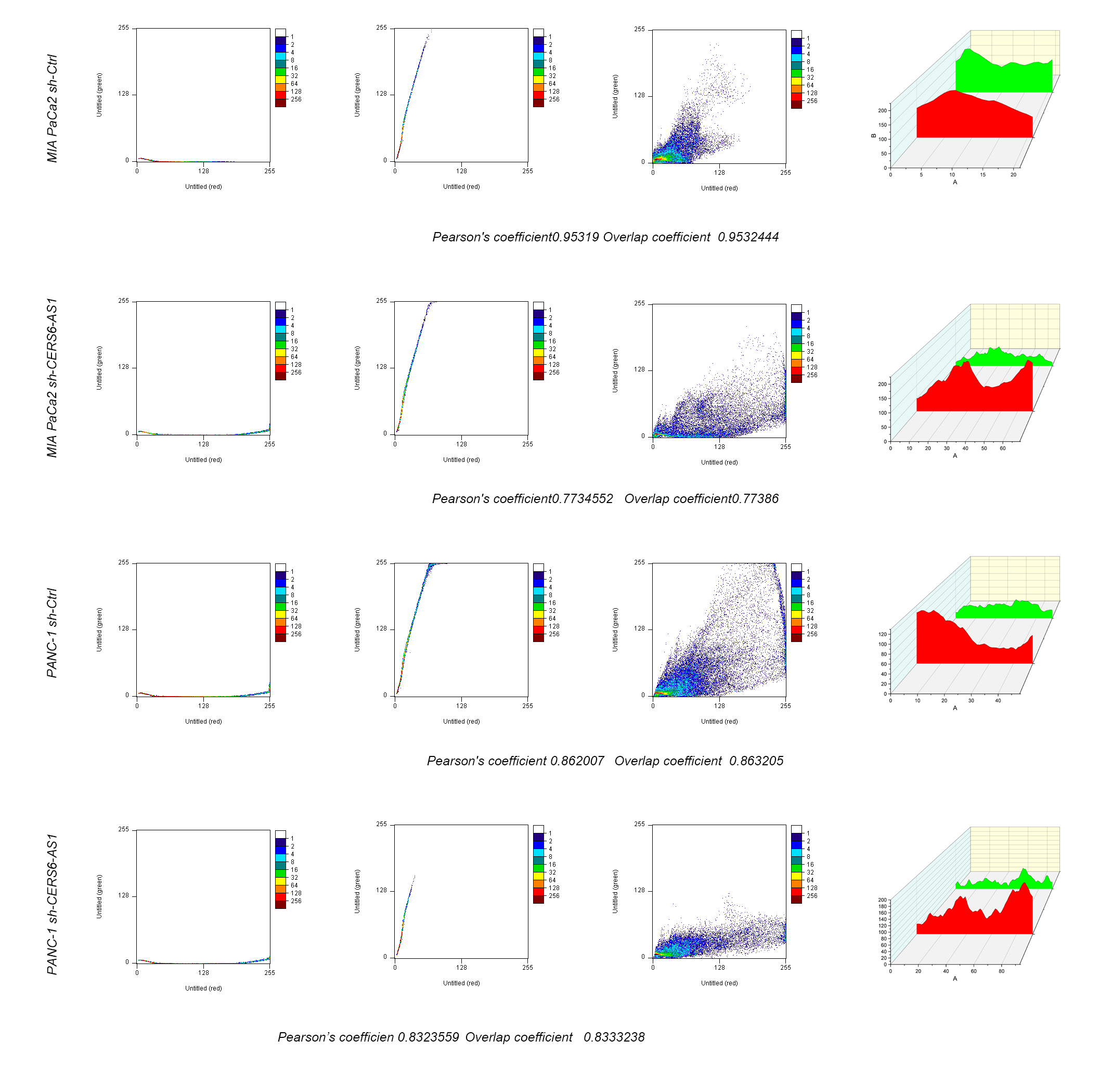

Supplement: Supplementary file 5 — supplemental Figure 3 [file 41419_2021_3921_MOESM5_ESM.tif]
